# Prevalence of Bacterial Urinary Tract Infections in Dogs and Cats with Lower Urinary Tract Diseases and Other Illnesses: A Systematic Review and Meta-Analysis

**DOI:** 10.3390/ani15233456

**Published:** 2025-11-30

**Authors:** Patchaya Thassakorn, Peerapol Sukon, Patchara Phuektes, Numfa Fungbun

**Affiliations:** 1 Faculty of Veterinary Medicine, Khon Kaen University, Khon Kaen 40002, Thailand; patchaya@kku.ac.th; 2 Division of Anatomy, Faculty of Veterinary Medicine, Khon Kaen University, Khon Kaen 40002, Thailand; sukonp@kku.ac.th; 3 Division of Pathobiology, Faculty of Veterinary Medicine, Khon Kaen University, Khon Kaen 40002, Thailand; patphu@kku.ac.th; 4 Division of Companion Animal Medicine, Faculty of Veterinary Medicine, Khon Kaen University, Khon Kaen 40002, Thailand

**Keywords:** urinary tract infections (UTIs), lower urinary tract diseases, prevalence, dogs, cats, systematic review, meta-analysis

## Abstract

Urinary tract infections (UTIs) occur in dogs and cats across diverse populations and regions. In this systematic review and meta-analysis, we estimated the pooled prevalence of UTIs in dogs and cats with lower urinary tract diseases (LUTDs) or other illnesses and characterized the distribution of bacterial uropathogens. A comprehensive search of PubMed, Scopus, and Web of Science identified 887 articles, of which 18 were published up to October 2024, met the inclusion criteria. Meta-analyses using a random-effects model estimated a pooled prevalence of 26.1%. Prevalence was greater in dogs (44.6%) than in cats (18.6%), and higher in females (30.1%) than in males (14.6%), affecting all age groups. Single-pathogen infections predominated, with *Escherichia coli* being the most common uropathogen in both species, followed by *Proteus* spp. and *Staphylococcus* spp. in dogs and *Staphylococcus* spp. in cats. Prevalence remained relatively constant over the study period (1991–2021). These findings underscore the value of pooled prevalence and bacterial distribution data for guiding empirical antimicrobial selection, and they highlight the need for further systematic reviews on antimicrobial susceptibility and multidrug resistance to inform treatment strategies.

## Simple Summary

Urinary tract infections (UTIs) are a significant health concern in dogs and cats and have public health implications, as companion animals can serve as reservoirs for pathogenic and multidrug-resistant (MDR) bacteria. In the study period, the estimated prevalence of UTIs in dogs and cats associated with LUTDs or other illnesses was 26.1%, with true values ranging from 8.0% to 58.9%. Prevalence was higher in dogs than cats and higher in females than in male, and all age groups were affected. *Escherichia coli* was the predominant causative bacterium, and prevalence remained stable over time.

## 1. Introduction

Urinary tract infections (UTIs) are a common clinical issue in dogs and cats and are generally classified as either simple (uncomplicated) or complicated UTIs. The former—also referred to as sporadic bacterial cystitis—are characterized by bacterial infection of the bladder that leads to inflammation and lower urinary tract signs, including pollakiuria, dysuria, hematuria, periuria, and urinary incontinence, in animals with otherwise normal urinary tract anatomy and function. In contrast, a complicated UTI is associated with anatomical or functional abnormalities of the urinary tract that predispose an animal to persistent or recurrent infections [1,2]. Subclinical bacteriuria is characterized by the isolation of bacteria via urine culture in the absence of clinical signs or cytological evidence of inflammation [1,2]. In more advanced cases, pyelonephritis may develop as a consequence of an ascending bacterial infection originating in the lower urinary tract [3].

Similarly to other infectious diseases, UTIs pose a significant public health concern, as dogs and cats can serve as reservoirs for pathogenic and multidrug-resistant (MDR) bacterial strains. These strains have the potential to be transmitted across species through close contact with household members, thereby increasing the risk of zoonotic infections in humans [4,5,6]. In addition, the overuse and misuse of antimicrobial agents have substantially contributed to the emergence and spread of resistant bacterial populations [7,8]. Consequently, newly emerged pathogenic strains exhibiting multidrug resistance may lead to therapeutic failure. This growing crisis underscores the urgent need for the development and discovery of novel antimicrobial agents to combat life-threatening infections [9].

The selection of antimicrobial therapy in clinical practice relies on clinical manifestations (e.g., sporadic bacterial cystitis and subclinical bacteriuria), bacterial culture, and antimicrobial susceptibility. Antimicrobials are the mainstay of treatment for sporadic bacterial cystitis; however, they are generally not recommended for subclinical bacteriuria [1]. Studies on the prevalence of UTIs contribute to public health awareness; additionally, information on the type and proportion of pathogenic bacteria causing UTIs, as well as patterns of antimicrobial susceptibility and resistance, is essential for developing empirical antimicrobial guidelines prior to the availability of culture and susceptibility results [10,11,12].

UTIs account for a substantial proportion of lower urinary tract diseases (LUTDs) in dogs and cats, and various other illnesses may predispose these animals to UTIs. Comorbidities associated with urinary tract infections include chronic kidney disease, hyperadrenocorticism, diabetes mellitus, and urinary bladder dysfunction secondary to thoracolumbar intervertebral disk disease [13,14,15,16,17]. However, the prevalence of UTIs in dogs and cats, their temporal trends, and the spectrum of causative uropathogens have not been comprehensively characterized. To address this gap, in the present study, we conducted a systematic review and meta-analysis of the population-based literature to estimate the prevalence of UTIs in dogs and cats with LUTDs or other conditions. Furthermore, we evaluated geographic distribution, species, sex, age, diagnostic thresholds, sampling methods, temporal trends, and the types and relative proportions of bacterial uropathogens.

## 2. Materials and Methods

### 2.1. Review Questions

The review questions were developed using the PECO framework [18], in which P denotes the study population, E the exposure of interest, C the comparator group, and O the disease outcome. Specifically, this review addressed the following questions: (1) What is the prevalence of bacterial UTIs in dogs and cats with LUTDs and other illnesses? (2) Which bacterial species are most frequently associated with for UTIs in these animal populations?

### 2.2. Search Strategy

**Figure 1:**
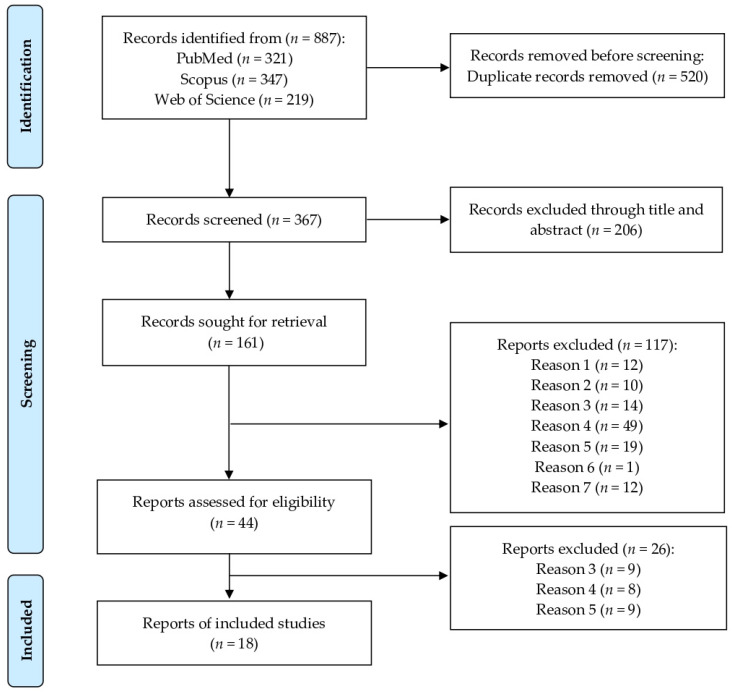
PRISMA flow diagram of the data search, extraction, and selection process. The literature search yielded 887 articles, of which 18 studies were included in the final review. The excluded studies were categorized as follows: **Reason 1**—studies that did not investigate naturally occurring bacterial UTIs; **Reason 2**—studies conducted on species other than dogs and cats; **Reason 3**—studies with no documented clinical signs related to urinary tract disease or studies focused exclusively on cases of subclinical bacteriuria; **Reason 4**—studies that did not report diagnostic testing for UTIs, lacked urine sample collection, or did not perform urine culture; **Reason 5**—studies with the following designs: randomized controlled trials, review articles, case reports, case series, case–control studies, editorials, conference abstracts, or short communications; **Reason 6**—articles not published in English; **Reason 7**—studies for which the full text was not available for download.

Our protocol was developed in accordance with the Preferred Reporting Items for Systematic Reviews and Meta-Analyses (PRISMA) guidelines. A comprehensive literature search was conducted across three electronic databases—PubMed, Scopus, and Web of Science—to identify relevant studies published up to October 2024, without restrictions on publication date. The search strategy employed Medical Subject Headings (MeSH) terms used individually or in combination as follows: (urinary tract infections OR cystitis OR pyelonephritis OR bacteriuria) AND (epidemiology OR prevalence OR incidence) AND (dogs OR canine OR cats OR feline). Data were extracted at the Faculty of Veterinary Medicine, Khon Kaen University, Thailand. All retrieved citations were systematically screened to determine their eligibility based on predefined inclusion criteria (Figure 1).

### 2.3. Eligibility Criteria

Eligible articles were initially screened by two independent reviewers based on their titles and abstracts. If the eligibility of a study could not be determined from this alone, the full text was retrieved for further assessment. Studies were included or excluded from this review based on whether they met the predefined eligibility criteria outlined in Table 1.

### 2.4. Data Extraction

Data were extracted from studies that fulfilled the eligibility criteria using standardized Microsoft Excel spreadsheets. The extracted variables encompassed the name of the first author, publication year, year of sample collection, study location (country and continent), journal of publication, study design, species of subjects, population characteristics, sampling method, detection technique and diagnostic criteria, age and gender of the study population, sample size, and number of identified UTIs cases.

### 2.5. Quality Assessment

The included studies were independently evaluated by two reviewers (R1 and R2) using a standardized critical appraisal tool, consisting of 9 questions and adapted from the JBI Critical Appraisal Checklist for Studies Reporting Prevalence Data [19]. The assessment criteria were as follows: Q1: Was the sample frame appropriate to address the target population? Q2: Were study participants sampled in an appropriate way? Q3: Was the sample size adequate? Q4: Were the study subjects and the setting described in detail? Q5: Was the data analysis conducted with sufficient coverage of the identified sample? Q6: Were valid methods used for the identification of the condition? Q7: Was the condition measured in a standard, reliable way for all participants? Q8: Was there appropriate statistical analysis? Q9: Was the response rate adequate, and if not, was the low response rate managed appropriately? To evaluate the methodological quality of each study, a scoring system was applied to each checklist item: Yes (score = 1), No (score = 0), and Unclear (score = 0). To ensure reliability, all studies were independently double assessed by the two reviewers. Any discrepancies were resolved through discussion until consensus; if agreement could not be achieved, a third reviewer was consulted. Inter-rater agreement was quantified using Cohen’s κ coefficient, which measures the level of agreement beyond chance. The strength of agreement was interpreted according to conventional guidelines: slight (κ = 0.00–0.20), fair (κ = 0.21–0.40), moderate (κ = 0.41–0.60), substantial (κ = 0.61–0.80), and almost perfect (κ = 0.81–1.00).

### 2.6. Statistical Analysis

All data extracted from the prepared Excel spreadsheet were imported into the Comprehensive Meta-Analysis (CMA) software, Biostat version 4 (Englewood, NJ, USA), for further statistical analysis. The logit transformation was applied to stabilize variance before pooling data from individual studies, defined as logit(p) = ln[p/(1 − p)], where p represents the proportion and ln denotes the natural logarithm [19]. The pooled prevalence of UTIs was then reported as a point estimate with a 95% confidence interval (CI), following back-transformation to enhance interpretability. The prediction interval (PI) was also calculated to evaluate the distribution of the true pooled prevalence in the population. A random-effects model was employed for all meta-analyses, based on the assumption of variability in settings among the included studies. *p* < 0.05 was considered statistically significant for all analyses, unless otherwise specified. Heterogeneity was assessed using Cochran’s Q statistic (Q test), while the I^2^ statistic was used to quantify the degree of heterogeneity. I^2^ values of 25.0%, 50.0%, and 75.0% were interpreted as representing low, moderate, and high heterogeneity, respectively [20]. In addition, a descriptive analysis was performed to summarize the prevalence (median and range) of bacterial isolates identified from urine cultures.

#### 2.6.1. Overall Meta-Analysis

To obtain an overall estimate of UTI prevalence, a meta-analysis was conducted combining data from dogs and cats, with individual studies used as the units of analysis to calculate the overall pooled prevalence.

#### 2.6.2. Subgroup Meta-Analysis

Subgroup analyses were conducted to explore potential sources of heterogeneity based on various study characteristics. For comparisons of cohort characteristics, studies were stratified into those involving UTIs with LUTDs and UTIs and other illnesses. UTI prevalence was also analyzed separately by region (Asia, Europe, and North America), species (dogs and cats), gender (male, female, and unspecified), age group (<10 years, ≥10 years, and unspecified), diagnostic threshold (10^3^ CFU/mL, and unspecified), and sampling method (cystocentesis and unspecified).

#### 2.6.3. Meta-Regression

Subgroup analysis was limited to one categorical variable at a time. Therefore, to explore potential sources of heterogeneity incorporating both categorical and continuous covariates in the prevalence of UTIs in dogs and cats, both univariable and multivariable meta-regression analyses were conducted. Although the results of the former were similar to those of the subgroup analysis, the results of the multivariable meta-regression accounted for the other confounding variables in the model. Covariates included in the analyses were the following: study year, cohort type, continent, species, sex, age, diagnostic threshold, and sampling methods. For each covariate, univariable meta-regression was used to estimate regression coefficients and crude odds ratios (ORs) with 95% confidence intervals (CIs). All covariates were then included in a multivariable meta-regression model to obtain coefficients and adjusted ORs. Statistical significance was set at *p* < 0.05.

### 2.7. Sensitivity Analysis

To evaluate the robustness of the pooled prevalence estimates for UTIs in dogs and cats, sensitivity analyses were conducted as follows. First, both fixed-effects and random-effects models were applied to examine their influence on the estimates. Second, a leave-one-out analysis sequentially excluded each study to assess the impact of individual studies on the overall results. Third, the effect of inclusion criteria was examined by comparing analyses including only UTIs and UTIs with illnesses versus those including all available data.

### 2.8. Publication Bias

Publication bias was evaluated using a funnel plot, complemented by formal statistical assessments employing Begg’s test [21] and Egger’s test [22]. A significance threshold of *p <* 0.05 was interpreted as indicative of potential publication bias. In instances of funnel plot asymmetry, the trim-and-fill method [23] was applied to estimate potentially missing studies and to provide an adjusted pooled prevalence.

## 3. Results

### 3.1. Literature Search and Study Characteristics

A total of 887 articles were identified through a systematic search of three electronic databases: PubMed (*n* = 321), Scopus (*n* = 347), and Web of Science (*n* = 219). After the removal of duplicate records, 367 articles were retained for further screening. Titles and abstracts were then reviewed, and studies were excluded if they did not meet the predefined inclusion criteria. As a result, 18 articles were deemed eligible and included in this meta-analysis [11,12,13,14,15,16,17,24,25,26,27,28,29,30,31,32,33,34]. Detailed characteristics of the included studies are presented in Table 2. Of the 18 included studies, 5 reported data on dogs, 10 on cats, and 3 on both dogs and cats. Additionally, 15 studies provided data on the prevalence of bacterial isolates identified from the urine of dogs and cats with UTIs.

### 3.2. Assessment of Study Quality

The methodological quality of the 18 included studies was appraised using the JBI critical appraisal checklist for prevalence research (Table 3). Overall, the studies demonstrated moderate to high methodological quality. All (100%) had an appropriate sample frame and adequate sample size and applied appropriate statistical analyses. Moreover, all reported valid and reliable methods for condition identification and measurement, although only 61% (11/18) fully met these criteria. Appropriate sampling methods were employed in 83% (15/18) of studies, while 67% (12/18) adequately described study subjects and settings. Data analyses were considered to sufficiently cover the identified sample in 61% (11/18) of the studies. The item concerning response rate was not applicable. All studies were independently assessed by two reviewers (R1 and R2) with complete data, and the inter-rater agreement was substantial (Cohen’s κ = 0.764; SE = 0.053; *t* = 12.697; *p* < 0.001).

### 3.3. Overall Prevalence Estimates

**Figure 2:**
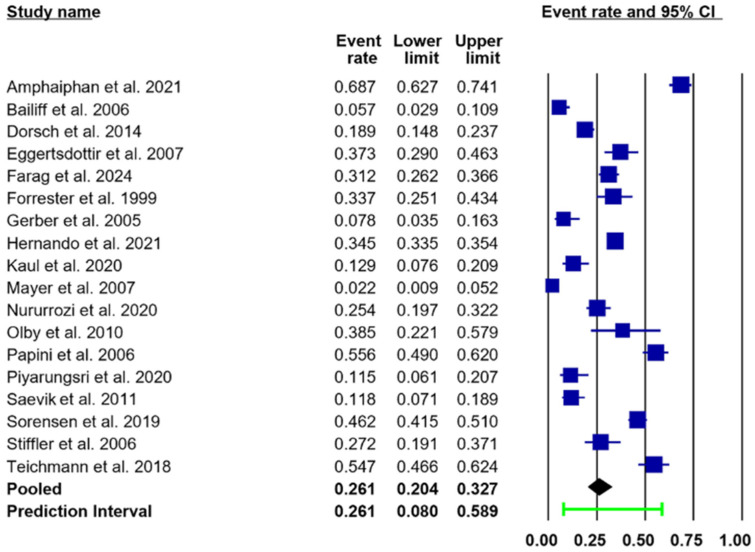
Forest plots illustrating an estimated pooled prevalence of UTIs (black diamond) of 26.1% (95% CI: 20.4–32.7%; PI: 8.0–58.9) in both dogs and cats [11,12,13,14,15,16,17,24,25,26,27,28,29,30,31,32,33,34]. Abbreviations: CI, confidence interval; PI, prediction interval (green line). The event rate and its 95% confidence interval (CI) for each study are represented by a navy-blue square.

**Figure 3:**
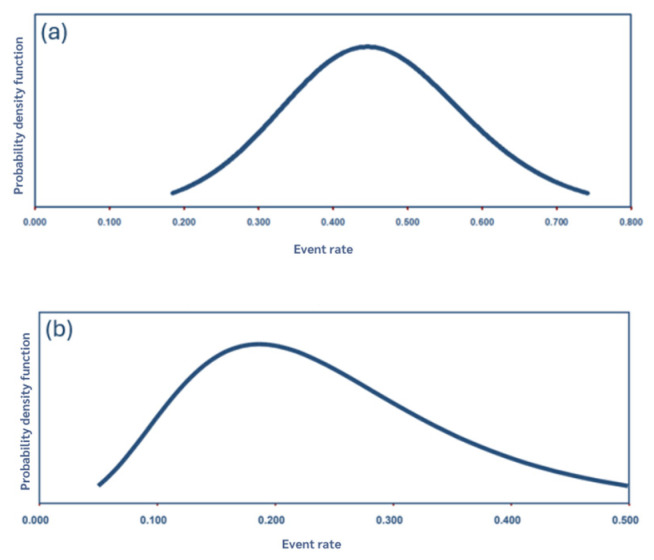
Distribution and point estimates of the pooled prevalence of UTIs in dogs (**a**) and cats (**b**).

Of the 18 included studies, the pooled prevalence of UTIs in both dogs and cats, estimated using a random-effects model, was 26.1% (95% CI: 20.4–32.7.9%; PI: 8.0–58.9), as illustrated in Figure 2 and Table 4. Distribution and point estimates of this prevalence are illustrated in Figure 3. A substantial level of heterogeneity was observed across the studies (I^2^ = 95.8%). Subgroup analysis suggested that variations in species and sex were the primary contributors to the observed heterogeneity.

### 3.4. Subgroup Analysis

#### 3.4.1. Cohort-Specific Prevalence

The pooled prevalence of UTIs in dogs and cats with LUTDs was 29.9% (95% CI: 23.9–36.8%; PI: 10.7–60.5), and that of UTIs with other illnesses was 20.1% (95% CI: 8.5–40.7%; PI: 0.6–90.6). However, this difference was not statistically significant (Table 4).

#### 3.4.2. Continent-Specific Prevalence

The prevalence of UTIs in dogs and cats was reported in 17 studies: 3 from Asia, 9 from Europe, and 5 from North America. Asia exhibited the highest pooled prevalence at 36.0% (95% CI: 12.5–68.9%; PI: 0.1–99.8), followed by Europe at 29.5% (95% CI: 22.7–37.2%; PI: 10.3–60.2) and North America at 15.3% (95% CI: 5.9–34.3%; PI: 0.3–91.4). However, these regional differences were not statistically significant (Table 4).

#### 3.4.3. Species-Specific Prevalence

The pooled prevalence of UTIs was 44.6% (95% CI: 36.0–53.6%; PI: 18.5–74.1) in dogs and 18.6% (95% CI: 13.6–25.0%; PI: 5.0–49.9) in cats; it was significantly higher in dogs than it was in cats (*p* < 0.001), as illustrated in Table 4.

#### 3.4.4. Sex-Specific Prevalence

Of the 18 included studies, 9 reported sex-specific data; females exhibited a significantly higher prevalence of UTIs than males, with pooled prevalence estimates of 30.1% (95% CI: 18.9–44.3%; PI: 5.3–76.8) and 14.6% (95% CI: 9.6–21.1%; PI: 3.3–46.5), respectively (*p* = 0.001). Gender-specific prevalence estimates are illustrated in Table 4.

#### 3.4.5. Age-Specific Prevalence

Of the 18 included studies, 6 reported age-specific data. The overall prevalence of UTIs was 19.6% (95% CI: 14.2–26.4%; PI: 0.3–95.8) in older animals (those aged ≥ 10 years), compared to 26.7% (95% CI: 17.0–39.2%; PI: 2.4–84.3) in younger and adult animals (aged < 10 years). However, this difference was not statistically significant (Table 4).

#### 3.4.6. Diagnostic Threshold Based on Quantitative Bacterial Cultures of Urine

Of the 18 studies included in this review, 11 defined a positive urine culture using a diagnostic cutoff of ≥10^3^ colony-forming units per milliliter (CFU/mL). Two studies employed higher values of ≥10^4^ CFU/mL and ≥10^5^ CFU/mL, respectively, while 5 studies did not report a specific diagnostic cutoff. The pooled prevalence of UTIs was 33.3% (95% CI: 22.9–45.0%; PI: 5.9–79.5) among studies applying the 10^3^ CFU/mL cutoff. Among research that did not specify a diagnostic criterion, the pooled prevalence was 14.5% (95% CI: 9.3–22.1%; PI: 2.9–49.0). Owing to the limited number of studies within certain subgroups, a comparative analysis of prevalence estimates across diagnostic thresholds was not feasible.

#### 3.4.7. Prevalence According to Sampling Methods

Of the 18 included studies, 16 reported sampling method data. The overall prevalence of UTIs was 29.4% (95% CI: 23.6–35.9%; PI: 10.1–60.5) with cystocentesis, compared to 19.2% (95% CI: 13.6–26.7%; PI: 0.2–96.7) with other/unspecified methods (Table 4).

### 3.5. Meta-Regression Analysis

Univariable meta-regression identified several study-level characteristics that were associated with the prevalence of UTIs in dogs and cats (Table 5). Species, sex, and diagnostic threshold demonstrated significant associations. Specifically, dogs had higher odds of UTIs compared with cats (OR = 1.51; 95% CI: 1.27–2.89; *p* < 0.001), and females were more likely to be affected than males (OR = 2.66; 95% CI: 1.38–5.16; *p* = 0.004). Studies that employed a diagnostic threshold of 10^3^ CFU/mL (OR = 2.72; 95% CI: 1.43–5.10; *p* = 0.002) or 10^4^ CFU/mL (OR = 3.35; 95% CI: 1.28–8.67; *p* = 0.014) reported significantly higher prevalence compared to those without a specified cutoff. In the multivariable model (Table 5), several factors remained significant after adjusting for potential confounders. Dogs continued to exhibit substantially higher odds of UTIs compared with cats (adjusted OR = 3.94; 95% CI: 2.32–6.69; *p* < 0.001). Similarly, female dogs and cats had significantly higher prevalence compared with males (adjusted OR = 2.64; 95% CI: 1.55–4.48; *p* < 0.001). Regarding continent, Europe reported a significantly higher prevalence compared with North America (adjusted OR = 6.82; 95% CI: 1.75–26.58; *p* = 0.006). In terms of clinical cohorts, the prevalence of UTIs among animals with other concurrent illnesses was significantly higher than those with UTIs with LUTDs (adjusted OR = 0.32; 95% CI: 0.10–0.97; *p* = 0.044). In the adjusted analysis, the diagnostic cutoff of 10^3^ CFU/mL remained significant (adjusted OR = 2.64; 95% CI: 1.39–4.95; *p* = 0.003), whereas the cutoff of 10^4^ CFU/mL did not. Overall, these findings suggest that species, sex, diagnostic criteria, clinical condition, and geographic region are important determinants of heterogeneity in the reported prevalence of UTIs in dogs and cats.

### 3.6. Sensitivity Analysis

The sensitivity analyses demonstrated that the pooled prevalence estimates were robust across model specifications and study selections. The fixed-effects model yielded 34.9% (95% CI: 34.1–35.9), and the random-effects model gave 26.1% (95% CI: 20.4–32.7). In the leave-one-out analysis, the pooled prevalence ranged from 24.2% (after removing the lowest prevalence [11]) to 28.7% (after removing the highest prevalence [13]), indicating that no single study unduly influenced the overall estimates. When examining the effect of inclusion criteria, the prevalence estimate was 26.1% (95% CI: 20.4–32.7) for studies including only UTIs associated with LUTDs or other illnesses and 26.1% (95% CI: 21.1–31.9) when all available data were included, further supporting the robustness of the findings (Table 6).

### 3.7. Prevalence of Pathogenic Bacteria Isolated from the Urine of Dogs and Cats with UTIs

*Escherichia coli* (median prevalence: 34.7%; range: 11.0–69.0%; eight studies), *Proteus* spp. (median: 12.6%; range: 2.4–16.6%; eight studies), and *Staphylococcus* spp. (median: 11.0%; range: 2.0–30.3%; seven studies) were the three most commonly identified bacterial species in canine UTIs. In feline cases, the two most frequently isolated bacteria were *E. coli* (median: 46.0%; range: 20.8–67.0%; nine studies) and *Staphylococcus* spp. (median: 10.7%; range: 4.2–33.3%; eight studies), as shown in Table 7 and Table 8.

### 3.8. Evaluation of Publication Bias

**Figure 4:**
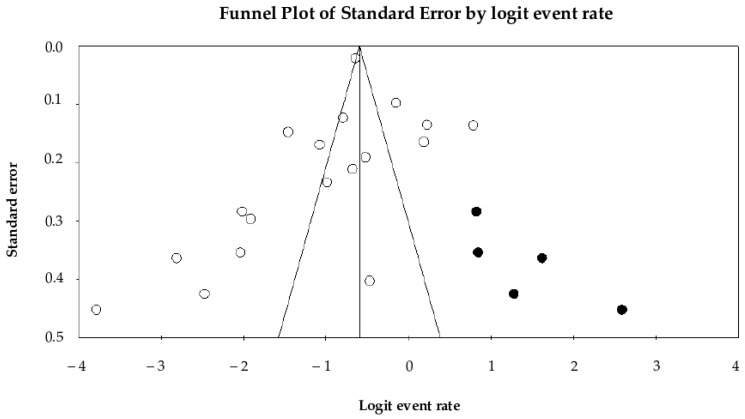
Visual inspection of the funnel plot reveals slight asymmetry, indicating potential publication bias. *X*-axis is the effect size (the logit of the event rate). *Y*-axis is the precision (standard error). Each included study is represented (white circle). Application of the trim-and-fill method suggested the presence of five potentially missing studies, which were imputed and are shown on the right side of the plot (black circles).

Begg’s test *(p* = 0.01) and Egger’s test (*p* = 0.35) evaluated potential publication bias. While Begg’s test indicated evidence of bias, Egger’s test did not reach statistical significance. Visual inspection of the funnel plot (Figure 4) reveals slight asymmetry; however, application of Duval and Tweedie’s trim-and-fill method identified five potentially missing studies. After imputing these studies, the estimated pooled prevalence of UTIs changed from 26.1% (95% CI: 20.4–32.7%) to 36.0% (95% CI: 29.0–43.6%).

## 4. Discussion

The pooled prevalence of UTIs in dogs and cats associated with LUTDs or other illnesses—including postoperative disk extrusion in dogs (two studies), hyperthyroidism in cats (one study), diabetes mellitus in cats (three studies), chronic kidney disease in cats (one study), and hyperadrenocorticism in dogs (one study)—was estimated at 26.1% using a random-effects meta-analysis. Substantial heterogeneity was observed (Q test, I^2^ = 95.8%; prediction interval: 8.0–58.9%), indicating considerable variability among the included studies [35]. The included studies differed in populations, disease associations, geographic regions, study periods, diagnostic thresholds, and sampling methods, all of which may have contributed to the high heterogeneity in the pooled estimates. Despite this variability, systematic reviews and meta-analyses provide important insights into disease burden, temporal patterns, and geographic distribution, supporting a broader understanding of UTIs in dogs and cats [36]. Heterogeneity in the pooled prevalence was explored using subgroup meta-analysis as well as univariate and multivariate meta-regression analyses. Seven potential sources of heterogeneity were examined, including study cohorts (UTIs associated with LUTDs vs. other illnesses), continents, species, sex, age, diagnostic thresholds, and sampling methods.

In our study, the estimated prevalence of UTIs associated with LUTDs or other illnesses did not differ significantly in the subgroup analyses (29.9% vs. 20.1%), although heterogeneity remained high. In the multivariate model, after adjustment for additional factors, the trend toward higher UTI prevalence in association with LUTDs versus other illnesses remained inconclusive, with a *p*-value (0.044) close to the conventional significance threshold.

Regarding geographic regions, the estimated prevalence of UTIs associated with LUTDs or other illnesses did not differ significantly among continents (36.0% in Asia, 29.5% in Europe, and 15.3% in North America). Africa was not included in the subgroup analysis because only a single study was available. High heterogeneity was observed within each continent, reflecting variability both between and within countries. For instance, in Asia, the estimated prevalence of UTIs in cats with LUTDs ranged from 40.8% in one study in Thailand [11] to 11.5% in another [25], with an intermediate estimate of 25.4% reported in Indonesia [26]. In Europe, prevalence estimates for cats with LUTDs were 54.7% in one study from Germany [29], 12.9% in another German study [27], and 7.8% in Switzerland [16]. However, according to multivariate meta-regression, after adjusting for other factors, the pooled prevalence of UTIs was significantly higher in Europe (adjusted OR = 6.82; 95% CI: 1.75–26.58; *p* = 0.006) compared with North America.

Regarding species, the estimated prevalence of UTIs associated with LUTDs or other illnesses was significantly higher in dogs than in cats (44.6% vs. 18.6%). After adjusting for other factors in multivariate meta-regression analyses, the pooled prevalence in dogs remained higher than that in cats (adjusted OR = 3.94; 95% CI: 2.32–6.69; *p* < 0.001). This finding aligns with previous reports from Thailand, Spain, and Egypt [11,12,24]. UTIs had the highest prevalence among dogs with lower urinary tract diseases, followed by those with micturition disorders, urolithiasis, prostatic diseases, and traumatic injuries [37,38]. In cats, studies have reported idiopathic cystitis as the most prevalent lower urinary tract disease, followed by urolithiasis and UTIs [16,25,27].

Regarding sex, female dogs and cats had a higher estimated prevalence of UTIs associated with LUTDs or other illnesses than males did (30.1% vs. 14.6%). Multivariate meta-regression analyses confirmed that this difference was significant (adjusted OR = 2.64; 95% CI: 1.55–4.48; *p* < 0.001), indicating that female dogs and cats are at greater risk. This difference is likely attributable to anatomical factors, as the shorter female urethra facilitates the ascending migration and colonization of uropathogenic bacteria in the urinary bladder [38]. Previous studies have also highlighted the role of fecal–perineal–urethral and urethral–urinary colonization, commensal flora, and other host factors in the pathogenesis of UTIs in both humans and dogs [39,40,41]. Infection typically begins when bacteria with virulence factors colonize the urethral epithelium and ascend to the bladder, especially in the presence of compromised host defenses or alterations in the vaginal microbiome, further explaining the higher prevalence in females [41].

Regarding age, the definition of a senior pet varies; for example, cats are generally considered senior at ≥10 years, whereas the threshold for dogs depends on breed, ranging from 6 to 7 years for large breeds to 11 years or older for small breeds. In this study, we used cutoffs of <10 years and ≥10 years. The estimated prevalence of UTIs associated with LUTDs or other illnesses did not differ significantly between these age groups (*p* = 0.231). Neither univariate nor multivariate meta-regression analyses indicated significant differences between the two groups. Although advancing age may increase susceptibility to infection—potentially due to immunosenescence, reduced mobility, and comorbid conditions [28,37]—our findings suggest that UTIs associated with LUTDs or other illnesses can affect dogs and cats across all age groups.

Regarding diagnostic thresholds, a quantitative urine culture with a cutoff of ≥10^3^ CFU/mL obtained via cystocentesis or catheterization is considered indicative of significant bacterial growth [42,43]. Subgroup meta-analysis showed that the estimated prevalence of UTIs was significantly higher in studies using a cutoff of 10^3^ CFU/mL compared with those with an unspecified diagnostic threshold (*p* = 0.004). After adjusting for other factors in multivariate analysis, the pooled prevalence remained higher for this value (adjusted OR = 2.64; 95% CI: 1.39–4.95; *p* = 0.003), whereas a cutoff of 10^4^ CFU/mL did not reach statistical significance (adjusted OR = 1.48; 95% CI: 0.59–3.67; *p* = 0.402). This pattern reflects the predominance of studies using the 10^3^ CFU/mL threshold for inclusion (11 studies with 10^3^ CFU/mL vs. 5 studies with unspecified thresholds).

Regarding urine sampling methods, studies using cystocentesis reported a significantly higher estimated prevalence of UTIs compared with those using other methods in subgroup analysis (*p* = 0.036). However, multivariate analysis showed no significant difference after adjusting for other factors (adjusted OR = 1.55; 95% CI: 0.73–3.29; *p* = 0.249).

The estimated prevalence of UTIs associated with LUTDs or other illnesses did not change significantly over time, according to both univariate and multivariate meta-regression analyses. These findings suggest a relatively constant prevalence of UTIs in dogs and cats with LUTDs and comorbid conditions over the study period. This stability is of public health concern, as dogs and cats can serve as reservoirs for pathogenic and multidrug-resistant bacterial strains.

Single bacterial infections were identified as a primary cause of UTIs in both dogs and cats. *E. coli* was the most frequently isolated pathogen, exhibiting the highest prevalence among UTI cases, a finding consistent with patterns observed in human clinical studies [44,45]. Uropathogenic *E. coli* (UPEC) refers to pathogenic strains of *E. coli* isolated from the urinary tract. UPEC has undergone adaptive evolution by producing a range of structural (fimbriae, pili, curli, and flagella) and secreted virulence factors, including toxins and iron-acquisition systems. These factors facilitate the bacterium’s ability to colonize, adhere to, and invade the urothelium, as well as to replicate intracellularly within host epithelial cells of the urinary tract [46,47]. The International Society for Companion Animal Infectious Diseases (ISCAID) recommended in 2019 that optimal empirical antimicrobial choices should be guided by pathogens and regional antimicrobial resistance patterns. Amoxicillin (AML) and trimethoprim–sulfamethoxazole (SXT) are recommended by ISCAID as first-tier options for the treatment of sporadic bacterial cystitis. Nitrofurantoin (NIT), fluoroquinolones, and third-generation cephalosporins may be effective when first-tier agents are deemed inappropriate based on culture and susceptibility testing results. Due to concerns regarding antimicrobial resistance and broader public health implications, fluoroquinolones are not recommended as first-line agents, particularly because they are excreted in active form in the urine, which may contribute to the development of resistant bacterial strains [1].

UPEC isolates from dogs exhibited susceptibility to amoxicillin–clavulanate (AMC) in 7.7–59.4% of cases, amoxicillin (AML) in 5–12.5%, cephalexin (CL) in 0–30.8%, enrofloxacin (ENR) in 25–44.4%, trimethoprim–sulfamethoxazole (SXT) in 15–53.8%, and nitrofurantoin (NIT) in 69.2%. Those from cats were susceptible to AMC in 25–80%, AML in 0%, CL in 14.3–34%, ENR in 0–44%, SXT in 20–71.4%, and NIT in 89.3% of cases [11,24,34,48]. *Staphylococcus* spp. isolates from dogs showed susceptibility to AMC in 20–87.9% of cases, AML in 33.3%, CL in 7–68.6%, ENR in 50–56.4%, and SXT in 20–30.2%. Meanwhile, those from cats were reported to be 100% susceptible to both AMC and ENR [11,16,48]. *Proteus* spp. isolates from dogs demonstrated susceptibility to AMC in 32–60% of cases, AML in 3%, CL in 15.4%, ENR in 11–36.7%, SXT in 33.3–63%, and NIT in 33.3%. Isolates from cats were susceptible to AMC in 75%, AML and CL in 0%, ENR in 50%, SXT in 50%, and NIT in 25% of cases [11,24]. Across these studies, UPEC, *Proteus* spp., and *Staphylococcus* spp. isolates from both dogs and cats exhibited greater susceptibility to AMC than to AML [11,48]. Moreover, in human medicine, a systematic review and meta-analysis reported a high resistance rate of 74.6% to AML among UPEC isolates [49]. In veterinary studies, the three major uropathogens demonstrated greater susceptibility to AMC compared to CL, ENR, and SXT. Similarly, a previous study found that the most frequently isolated uropathogenic species in dogs and cats exhibited susceptibility rates of 93.4% to AMC, 87.6% to SXT, and 76.1% to AML [50]. However, these findings were derived from a small-scale investigation. Overall, UPEC isolates obtained from canine and feline urine samples have demonstrated MDR rates ranging from 11.9% to 55.2% [50,51]. The high resistance rates observed among uropathogens are an increasing concern, particularly given the potential for bidirectional transmission of MDR bacterial strains between companion animals and humans. A large-scale systematic review and meta-analysis examining antimicrobial susceptibility and resistance patterns would provide more robust and generalizable evidence to guide the development of effective, evidence-based antimicrobial stewardship strategies for the management of UTIs in dogs and cats. Data on the patterns of uropathogens causing UTIs in dogs and cats, together with their drug susceptibility profiles, provide valuable insights with potential relevance to the human population. Many bacterial species implicated in veterinary UTIs overlap with those causing infections in humans, and similarities in resistance mechanisms underscore the interconnectedness of antimicrobial resistance across species. Such information is particularly important in the context of rising bacterial resistance and the documented misuse of antibiotics, in both human and veterinary medicine. By identifying resistance trends and highlighting the consequences of inappropriate antibiotic use, these findings reinforce the need for responsible prescribing practices, coordinated surveillance, and the adoption of a One Health approach to mitigate the global threat of antimicrobial resistance.

Publication bias is a well-documented concern in systematic reviews and meta-analyses. Several factors contribute to this bias, including the exclusion of studies published in different languages, the inclusion of studies with small sample sizes, and the preferential publication of studies reporting either extremely high or very low prevalence estimates. In particular, this study has several limitations that should be acknowledged. First, the scope of this meta-analysis was restricted to English-language publications and three electronic databases, which may have resulted in the omission of relevant studies and introduced language and selection bias. Second, considerable heterogeneity and evidence of publication bias were observed. Heterogeneity refers to the variability among studies included in a systematic review and meta-analysis, which may arise from differences in study populations, outcome measures, and methodological designs [52]. In this study, the high level of heterogeneity is likely attributable to such variations. The presence of substantial heterogeneity can limit the interpretability and generalizability of the pooled results, potentially reducing their applicability to a broader population. This limitation justifies the implementation of subgroup analyses to explore sources of variation. Third, certain data could not be included in subgroup analyses due to an insufficient number of studies in some categories, resulting in limited comparative power. Given these limitations, the prevalence estimates presented in this study should be interpreted as relative.

## 5. Conclusions

This systematic review and meta-analysis used a random-effects model to estimate the pooled prevalence of UTIs in dogs and cats associated with LUTDs or other illnesses, which was 26.1%, with high heterogeneity. The distribution of true prevalence ranged from 8.0% to 58.9%. The pooled prevalence was higher in Europe than in North America. UTIs were more prevalent in dogs and in females, although they affected animals across all age groups. The estimated prevalence did not change significantly over time. Single-species bacterial infections, particularly those caused by *E. coli*, accounted for the majority of cases in both species. These observed patterns in uropathogen type may inform more targeted empirical antimicrobial selection in clinical veterinary practice. Future large-scale systematic reviews and meta-analyses focusing on antimicrobial susceptibility and MDR patterns among uropathogens are warranted. Trends in such pathogens and their drug resistance profiles highlight shared antimicrobial risks across species, emphasizing the urgent need for responsible antibiotic use and a One Health approach to combat rising resistance.

## Figures and Tables

**Table 1:** Inclusion and exclusion criteria of this study.

Inclusion Criteria	Exclusion Criteria
1. Studies investigating naturally occurring bacterial urinary tract infections (UTIs), defined as the presence of clinical signs consistent with lower urinary tract diseases (LUTDs) in combination with a positive urine culture.	1. Studies that did not investigate naturally occurring bacterial urinary tract infections (UTIs), for example, those involving experimentally induced UTIs or in vitro models.
2. Studies conducted on canine and feline species.	2. Studies conducted on species other than dogs and cats.
3. Studies reporting the prevalence of UTIs, including the total population examined and the number of positive urine cultures.	3. Studies focusing exclusively on cases of subclinical bacteriuria.
4. Studies that were cross-sectional and observational in design, either retrospective or prospective.	4. Studies that did not report diagnostic testing for UTIs, lacked urine sample collection, or did not perform urine culture.
5. Articles published in English.	5. Studies employing the following designs: randomized controlled trials, review articles, case reports, case series, case–control studies, editorials, conference abstracts, or short communications.
6. Full-text articles available from online databases.	6. Articles not published in English.
	7. Full-text articles not available for download.

**Table 2:** Characteristics of the 18 studies included in this meta-analysis.

Author	Country	Species	Condition	Diagnostic Cutoff (CFU/mL)	Prevalence Rate (%)
[11]	Thailand	dogs	With urologic problems	10^3^	75.37
[11]	Thailand	cats	With urologic problems	10^3^	40.82
[12]	Spain	dogs	With clinical signs of LUTDs	NA	39.09
[12]	Spain	cats	With clinical signs of LUTDs	NA	24.62
[13]	The United States of America	cats	Hyperthyroidism, diabetic mellitus, and chronic kidney disease	NA	2.23
[14]	The United States of America	cats	Diabetic mellitus and clinical signs of lower urinary tract disease	10^3^	5.67
[15]	The United States of America	dogs	After surgical intervertebral disk extrusion	10^5^	27.17
[16]	Switzerland	cats	With clinical signs of LUTDs	NA	7.79
[17]	The United States of America	dogs	Diabetic mellitus or hyperadrenocorticism with signs of LUTD	10^3^	33.66
[24]	Egypt	dogs	With clinical signs of LUTDs	10^3^	36.99
[24]	Egypt	cats	With clinical signs of LUTDs	10^3^	25.93
[25]	Thailand	cats	With clinical signs of LUTDs	NA	11.54
[26]	Indonesia	cats	With clinical signs of LUTDs	10^3^	25.41
[27]	Germany	cats	LUTDs	NA	12.87
[28]	Denmark	dogs	With clinical signs of LUTDs	10^4^	46.23
[29]	Germany	cats	With clinical signs of LUTDs	10^3^	54.67
[30]	Germany	cats	With clinical signs of LUTDs	10^3^	18.87
[31]	Norway	cats	With clinical signs of LUTDs	10^3^	11.76
[32]	The United States of America	dogs	After surgical disk extrusion	10^3^	38.46
[33]	Norway	cats	With clinical signs of LUTDs	10^3^	37.29
[34]	Italy	dogs	With clinical signs suggestive of UTIs	10^3^	55.61

LUTDs: lower urinary tract diseases; NA: not applicable.

**Table 3:** The study quality assessment presents the number of included studies within each category of a simplified rating scale, based on a nine-item evaluation checklist.

Items	No. of Included Studies in Each Category			
	Yes (Score = 1)	No (Score = 0)	Unclear (Score = 0)	Total Score
1. Was the sample frame appropriate to address the target population?	18	0	0	18/18
2. Were study participants sampled in an appropriate way?	15	3	0	15/18
3. Was the sample size adequate?	18	0	0	18/18
4. Were the study subjects and the setting described in detail?	12	6	0	12/18
5. Was the data analysis conducted with sufficient coverage of the identified sample?	11	7	0	11/18
6. Were valid methods used for the identification of the condition?	11	7	0	11/18
7. Was the condition measured in a standard, reliable way for all participants?	11	7	0	11/18
8. Was there appropriate statistical analysis?	18	0	0	18/18
9. Was the response rate adequate, and if not, was the low response rate managed appropriately?	NA	NA	NA	NA

NA: not applicable.

**Table 4:** Overall and subgroup meta-analyses of the prevalence of urinary tract infections in dogs and cats with lower urinary tract diseases and other illnesses.

Categories	No. of Studies or Subgroups	Prevalence (%)			Heterogeneity			*p*-Value for Subgroup Difference
		Estimate	95% CI	PI	Q	*p*-Value	I^2^ (%)	
Overall	18	26.1	20.4–32.7	8.0–58.9	402.4	<0.001	95.8	
Cohort								0.322
UTIs with LUTDs	12	29.9	23.9–36.8	10.7–60.5	498.0	<0.001	97.2	
UTIs with other illnesses	6	20.1	8.5–40.7	0.6–90.6	111.1	<0.001	95.5	
Continent ^a^								0.295
Asia	3	36.0	12.5–68.9	0.1–99.8	119.6	<0.001	97.5	
Europe	9	29.5	22.7–37.2	10.3–60.2	378.3	<0.001	97.6	
North America	5	15.3	5.9–34.3	0.3–91.4	62.0	<0.001	93.5	
Species								<0.001
Dogs	8	44.6	36.0–53.6	18.5–74.1	126.3	<0.001	94.5	
Cats	13	18.6	13.6–25.0	5.0–49.9	175.5	<0.001	93.2	
Sex								0.001
Female	9	30.1	18.9–44.3	5.3–76.8	56.6	<0.001	85.9	
Male	9	14.6	9.6–21.1	3.3–46.5	51.7	<0.001	84.5	
Unspecified	9	36.3	26.6–47.2	10.0–74.6	223.2	<0.001	96.4	
Age								0.231
<10 years	4	26.7	17.0–39.2	2.4–84.3	44.5	<0.001	93.3	
≥10 years	3	19.6	14.2–26.4	0.3–95.8	11.5	<0.001	82.6	
Unspecified	12	28.8	19.8–39.9	5.0–75.8	288.5	<0.001	95.5	
Diagnostic threshold ^b^								0.004
10^3^ CFU/mL	11	33.3	22.9–45.0	5.9–79.5	278.3	<0.001	98.5	
Unspecified	5	14.5	9.3–22.1	2.9–49.0	298.4	<0.001	97.6	
Sampling method								0.036
Cystocentesis	9	29.4	23.6–35.9	10.1–60.5	551.2	<0.001	96.9	
Other	9	19.2	13.6–26.7	0.2–96.7	6.9	<0.001	71.0	

^a^ Africa was excluded from the analysis due to the low number of studies or subgroups (*n* = 1). ^b^ Cutoffs at 10^4^ and 10^5^ CFU/mL were excluded from the analysis due to the low number of studies or subgroups (*n* = 2). Abbreviations: CI, confidence interval; PI, prediction interval; CFU/mL, colony-forming unit/mL.

**Table 5:** Univariable and multivariable meta-regression analyses of the prevalence of urinary tract infections in dogs and cats with clinical conditions.

Categories	Univariable Meta-Regression			Multivariable Meta-Regression		
	Coefficient (95% CI)	Crude OR (95% CI)	*p*-Value	Coefficient (95% CI)	Adjusted OR (95% CI)	*p*-Value
Study year	0.02 (−0.02–0.05)	1.02 (0.98–1.05)	0.310	0.00 (−0.06–0.06)	1.00 (0.94–1.06)	0.922
Cohort						
UTIs with LUTDs	0.17 (−0.41–0.75)	1.19 (0.66–2.12)	0.570	−1.13 (−2.22–−0.03)	0.32 (0.10–0.97)	0.044
UTIs with other illnesses	Reference			Reference		
Continent						
Africa	0.53 (−0.54–1.59)	1.70 (0.18–4.90)	0.331	1.35 (−0.94–3.64)	3.86 (0.39–38.09)	0.248
Asia	0.52 (−0.30–1.34)	1.68 (0.74–3.82)	0.210	2.59 (0.59–4.59)	13.33 (1.80–98.49)	0.011
Europe	0.41 (−0.24–1.06)	1.51 (0.79–2.89)	0.215	1.92 (0.56–3.28)	6.82 (1.75–26.58)	0.006
North America	Reference			Reference		
Species						
dogs	1.18 (0.66–1.69)	1.51 (1.27–2.89)	<0.001	1.37 (0.84–1.90)	3.94 (2.32–6.69)	<0.001
cats	Reference			Reference		
Sex						
Female	0.98 (0.32–1.64)	2.66 (1.38–5.16)	0.004	0.97 (0.44–1.50)	2.64 (1.55–4.48)	<0.001
Unspecified	1.18 (0.61–1.76)	3.25 (1.84–5.81)	<0.001	0.69 (0.05–1.33)	1.99 (1.05–3.78)	0.035
Male	Reference			Reference		
Age						
<10 years	0.30 (−0.68–1.28)	1.35 (0.51–3.60)	0.551	−0.63 (−1.37–0.12)	0.53 (0.25–1.13)	0.100
Unspecified	0.50 (−0.35–1.34)	1.65 (0.70–3.82)	0.248	−0.53 (−1.31–0.25)	0.59 (0.27–1.28)	0.184
≥10 years	Reference			Reference		
Diagnostic threshold						
10^3^ CFU/mL	1.00 (0.36–1.63)	2.72 (1.43–5.10)	0.002	0.97 (0.33–1.60)	2.64 (1.39–4.95)	0.003
10^4^ CFU/mL	1.21 (0.25–2.16)	3.35 (1.28–8.67)	0.014	0.39 (−0.52–1.30)	1.48 (0.59–3.67)	0.402
Unspecified	Reference			Reference		
Sampling method						
Cystocentesis	0.59 (−0.07–1.25)	1.80 (0.93–3.49)	0.079	0.44 (−0.31–1.19)	1.55 (0.73–3.29)	0.249
Other	Reference			Reference		

Abbreviations: CI, confidence interval; OR, odds ratio; CFU/mL, colony-forming unit/mL.

**Table 6:** Sensitivity analysis of the robustness of the estimates for the prevalence of urinary tract infections in dogs and cats with lower urinary tract diseases and other illnesses.

Categories	No. of Studies or Subgroups	Prevalence (%)	
		Estimate	95% CI
Model			
Fixed effects	18	34.9	34.1–35.9
Random effects	18	26.1	20.4–32.7
Leave-one-out analysis			
Lowest prevalence ^a^	17	24.2	19.2–30.1
Highest prevalence ^b^	17	28.7	22.8–35.3
Inclusion criteria			
UTIs with LUTDs or UTIs with other illnesses	18	26.1	20.4–32.7
All data	22	26.1	21.1–31.9

^a^ The study with the lowest prevalence was removed [11]. ^b^ The study with the highest prevalence was removed [13]. Abbreviations: UTIs, urinary tract infections; LUTDs, lower urinary tract diseases; CI, confidence interval.

**Table 7:** Proportion of bacterial isolates identified in dogs with UTIs.

Bacteria	Author								Mean ± SD	Median	Min	Max
	[11]	[12]	[15]	[17]	[24]	[28]	[32]	[34]				
**Single infection**	-	89.2	90	83.3	-	-	66.6	-	82.3 ± 10.9	86.3	66.6	90.0
*E. coli*	16.7	45.3	11	69	46.4	24	58.3	17.5	36.0 ± 21.6	34.7	11.0	69.0
*Proteus* spp.	13.6	13.2	4.5	2.4	16.1	12	8.3	16.6	10.8 ± 5.3	12.6	2.4	16.6
*Pseudomonas* spp.	13.1	4.1	-	-	12.5	-	-	7.2	10.0 ± 4.3	9.9	4.1	13.1
*Klebsiella* spp.	7.1	5.1	-	14.3	5.3	2	25	0.9	8.5 ± 8.5	5.3	0.9	25.0
*Enterobacter* spp.	0.5	1.7	-	7.1	3.6	-	-	-	3.2 ± 2.9	2.7	0.5	7.1
*Pasteurella* spp.	-	0.1	-	-	-	-	-	-	-	-	0.1	0.1
*Staphylococcus* spp.	30.3	11	13.6	4.8	-	2	16.6	6.7	12.1 ± 9.5	11.0	2.0	30.3
*Enterococcus* spp.	5.1	8.6	12	7.1	10.7	-	25	-	11.4 ± 7.1	9.7	5.1	25.0
*Streptococcus* spp.	9.6	6.5	-	14.3	-	1	8.3	2.2	7.0 ± 4.9	7.4	1.0	14.3
*Bacillus* spp.	0.5	0.1	-	-	-	-	-	-	0.3 ± 0.3	0.3	0.1	0.5
Other genera	3	4.3	4.5	7.1	-	2	-	-	4.2 ± 1.9	4.3	2.0	7.1
**Mixed infection**	-	10.8	10	16.6	3.7	-	33.3	-	14.9 ± 11.3	10.8	3.7	33.3
Two organisms	-	-	-	-	100	-	75	-	87.5 ± 17.7	87.5	75.0	100.0
Three organisms	-	-	-	-	-	-	25	-	-	-	25.0	25.0
Four organisms	-	-	-	-	-	-	-	-	-	-	0.0	0.0
**Fungal infection**	-	0.4	-	-	-	-	-	-	-	-	0.4	0.4

**Table 8:** Proportion of bacterial isolates identified in cats with UTIs.

Bacteria	Author									Mean ± SD	Median	Min	Max
	[11]	[12]	[13]	[14]	[16]	[29]	[30]	[31]	[33]				
**Single infection**	-	87	-	88.9	-	-	-	-	72.3	82.7 ± 9.1	87.0	72.3	88.9
*E. coli*	20.8	42.7	46	67	66.7	50.5	65.3	44.4	38.5	49.1 ± 15.3	46.0	20.8	67.0
*Proteus* spp.	16.7	3.4	2.7	-	-	2.6	-	5.5	1.5	5.4 ± 5.7	3.1	1.5	16.7
*Pseudomonas* spp.	25	4.2	5.4	-	-	-	-	5.5	-	10.0 ± 10.0	5.5	4.2	25.0
*Klebsiella* spp.	12.5	2.5	-	5	-	-	-	-	-	6.7 ± 5.2	5.0	2.5	12.5
*Enterobacter* spp.	-	2.6	2.7	5	-	-	-	-	3	3.3 ± 1.1	2.9	2.6	5.0
*Pasteurella* spp.	4.2	0.4	2.7	-	-	-	-	5.6	4.6	3.5 ± 2.0	4.2	0.4	5.6
*Staphylococcus* spp.	4.2	15.2	5.4	-	33.3	22.9	10.2	11.1	9.2	13.9 ± 9.8	10.7	4.2	33.3
*Enterococcus* spp.	8.3	22.2	27	5	-	15.1	4.1	11.1	3	12.0 ± 8.8	9.7	3.0	27.0
*Streptococcus* spp.	4.2	2	5.4	17	-	3.6	8.2	5.5	6.1	6.5 ± 4.6	5.5	2.0	17.0
*Bacillus* spp.	-	0.1	-	-	-	-	-	-	-	-	-	0.1	0.1
Other genera	4.1	4.7	2.7	-	33.3	-	12.3	11.1	6.1	10.6 ± 10.6	6.1	2.7	33.3
**Mixed infection**	-	13	-	11.1	-	-	-	-	27.6	17.2 ± 9.0	13.0	11.1	27.6
**Fungal infection**	-	0.9	-	-	-	-	-	-	-	-	-	0.9	0.9

## Data Availability

Data are contained within this article.
